# Comparative genome analysis of *Erysipelothrix rhusiopathiae* isolated from domestic pigs and wild boars suggests host adaptation and selective pressure from the use of antibiotics

**DOI:** 10.1099/mgen.0.000412

**Published:** 2020-07-31

**Authors:** Robert Söderlund, Nicoletta Formenti, Stefania Caló, Mario Chiari, Mate Zoric, Giovanni Loris Alborali, Tina Sørensen Dalgaard, Eva Wattrang, Helena Eriksson

**Affiliations:** ^1^​ National Veterinary Institute (SVA), Uppsala, Sweden; ^2^​ Istituto Zooprofilattico Sperimentale della Lombardia e Dell'Emilia Romagna, Brescia, Italy; ^3^​ Aarhus University, Aarhus, Denmark

**Keywords:** erysipelas, wildlife, genomics, antibiotic resistance, molecular epidemiology, pan-genome analysis

## Abstract

The disease erysipelas caused by *Erysipelothrix rhusiopathiae* (ER) is a major concern in pig production. In the present study the genomes of ER from pigs (*n*=87), wild boars (*n*=71) and other sources (*n*=85) were compared in terms of whole-genome SNP variation, accessory genome content and the presence of genetic antibiotic resistance determinants. The aim was to investigate if genetic features among ER were associated with isolate origin in order to better estimate the risk of transmission of porcine-adapted strains from wild boars to free-range pigs and to increase our understanding of the evolution of ER. Pigs and wild boars carried isolates representing all ER clades, but clade one only occurred in healthy wild boars and healthy pigs. Several accessory genes or gene variants were found to be significantly associated with the pig and wild boar hosts, with genes predicted to encode cell wall-associated or extracellular proteins overrepresented. Gene variants associated with serovar determination and capsule production in serovars known to be pathogenic for pigs were found to be significantly associated with pigs as hosts. In total, 30 % of investigated pig isolates but only 6 % of wild boar isolates carried resistance genes, most commonly *tetM* (tetracycline) and *lsa*(E) together with *lnu*(B) (lincosamides, pleuromutilin and streptogramin A). The incidence of variably present genes including resistance determinants was weakly linked to phylogeny, indicating that host adaptation in ER has evolved multiple times in diverse lineages mediated by recombination and the acquisition of mobile genetic elements. The presented results support the occurrence of host-adapted ER strains, but they do not indicate frequent transmission between wild boars and domestic pigs. This article contains data hosted by Microreact.

## Data Summary

Sequence data have been uploaded to the European Nucleotide Archive (https://www.ebi.ac.uk/ena) together with isolate metadata (host, year and country of origin) and are available under project accession number PRJEB34817. The FASTA format pan-genome analysis output and the filtered Scoary pan-genome-wide association analysis output are presented as data file 1 and Table S1 (available in the online version of this article) respectively. *In silico* serotyping results are presented in Table S2. An interactive presentation of the relationship between host type, SNP data, selected genetic resistance determinants and *in silico* serotyping results for pig and wild boar isolates is available via Microreact at https://microreact.org/project/wLkvdEnp9iNXMJhGb3bgxZ?tt=rd&fc=Host

**Impact Statement**

*
Erysipelothrix rhusiopathiae* (ER) is a bacterial pathogen that can infect several species including humans but is a particular economic and animal welfare problem in pig farming. Previous studied have shown that certain types of ER cause most of the severe outbreaks among pigs. A growing trend of free-range animal husbandry and expanding wild boar populations in Europe raises the question of whether strains of ER adapted to infect pigs are spread by wild boars. In the present study we have compared ER from pigs and wild boars in Sweden, Denmark and Italy using whole-genome genetic methods. Our results support the suggestion that certain strains of ER are adapted to pigs and wild boars and defines genetic markers to identify such adapted strains. We also show that genes providing resistance to antibiotics are relatively common among ER from pigs but not among ER from wild boars. Our results indicate that infection spread between wild boars and pigs could probably cause severe disease, which has implications for farm biosecurity measures, but we see no indication that such transmission is common on the national or regional level.

## Introduction

The Gram-positive bacterium *Erysipelothrix rhusiopathiae* (ER) is the causative agent of the disease erysipelas in several warm-blooded animal species [1]. Although well known as an economically important pathogen in pig and poultry farming, ER occurs as a pathogen or commensal in a wide variety of other species [1]. Compared to other non-spore-forming bacteria, ER is well adapted to survive in the environment, and can persist for weeks in soil especially at lower temperatures [2]. Erysipelas in pigs can manifest in an acute septicaemic form with characteristic red diamond-shaped skin lesions, a clinically less severe subacute form with fever, depression and sometimes skin lesions, or as chronic conditions including endocarditis or arthritis [3]. Many pigs (up to 30–50 % in some populations) carry and shed the bacteria for extended periods without showing signs of disease [3]. Infection can be prevented by vaccination or treated with antibiotics, but ER still has substantial economic impact on the pig production industry through treatment and vaccination costs, growth rate reduction, animal losses and costs associated with rejection at slaughter [4, 5]. ER is also a zoonotic pathogen; handling of infected animals or meat can cause localized skin infection in exposed workers such as butchers and veterinarians, a condition referred to as erysipeloid [6]. More severe forms of ER infections in humans also occur, including endocarditis and septicaemia [6].

Consumer preferences in Europe and elsewhere are promoting an expansion of free-range production systems with access to both indoor and outdoor areas for food-producing animals. This leads to an increased exposure to the environmental bacterial flora and possibly to exchange of pathogens with wildlife. As the wild boar and the domestic pig are the same species (*Sus scrofa*), exchange of host-adapted strains of ER could be a concern considering the rapidly expanding wild boar populations in many European countries [7]. Several serological studies have indicated that ER infection is common among wild boars [8–11], and outbreaks of acute erysipelas have been reported among farmed wild boars [12, 13]. These outbreaks have generally been caused by the ER serovars 1a, 1b or 2, which are the same serovars that cause the majority of cases of acute and subacute erysipelas among domestic pigs [3], supporting the suggestion of particular strains being more virulent or better adapted to infect pigs and wild boars. Such adaptive processes are known to frequently involve the acquisition of mobile genetic elements that increase host- or niche-specific fitness [14] but also the loss of genes or gene function via mutations to adapt metabolic pathways and regulatory processes [15]. Genes encoding proteins that are released into the extracellular environment or displayed on the cell wall allow bacteria to directly interact with host tissues and the host immune system [16], and may therefore be expected to play a key role in host adaptation. However, a recent genomics study on ER divided the species into three generalist clades (1, 2, 3) and found little evidence of geographical or host-related genetic bias with the exception of clade 1, which was not observed in pigs, instead occurring among marine mammals, fish and a few wildlife samples [17].

The genomic characteristics of selected ER isolates derived from pigs have been studied extensively in recent years [17–21], but no comprehensive study of isolates from wild boars or comparison between ER isolated from wild boars and domestic pigs has been performed to date. In the present study we have used whole-genome sequencing to investigate a large panel of isolates of ER from domestic pigs in Denmark, Italy and Sweden and compared them to wild boar isolates of ER from Italy and Sweden (Denmark has no resident wild boar population) as well as isolates from other types of animals. The aim was to relate isolate characteristics in terms of phylogenetic relatedness and the presence of genes and gene variants to isolate sources in terms of host and country of origin. Such information is of importance to estimate the risk of ER transmission between free-range pigs and wild boars, and to better understand the evolution of ER in response to the biology of the porcine hosts and to animal husbandry practices.

## Methods

### Bacterial isolates and reference data

Isolates of ER from domestic pigs diagnosed with erysipelas in Sweden, Italy and Denmark between 1987 and 2018 were held in storage at −70 °C at the National Veterinary Institute (SVA), Sweden, the Istituto Zooprofilattico Sperimentale della Lombardia e Dell'Emilia Romagna (IZSLER), Italy, and the Danish Pig Research Centre (SEGES), Denmark. Additional isolates were collected in a slaughterhouse prevalence study performed on tonsils collected from 200 healthy pigs in Sweden 2017 [22] and in studies collecting ER isolates from the tonsils of healthy wild boars, performed in collaboration with hunters in the Swedish county of Östergötland and the Lombardy region of Italy in 2017–2018 (N. Formenti *et al*., manuscript in preparation). A total of 72 isolates from pigs and 71 from wild boars were included in the study. Limited information was in general available for the historical isolates beyond host animal type, year and country of origin. The genome sequences of 100 further isolates of ER from a wide variety of animal sources from the work of Forde *et al*. [17] were retrieved from GenBank and included for comparison. Sequences with high coverage were preferentially selected. The set consisted of isolates from wild ungulates (*n*=59), pigs (*n*=15), poultry (*n*=13), fish (*n*=5), marine mammals (*n*=4) and other sources (*n*=4).

### Whole-genome sequencing

DNA was extracted from bacterial isolates using an automated Magnetic Biosolutions Magnatrix 8000+ system run with a Diasorin Bullet Stool Kit. Sequencing libraries were prepared using the Illumina Nextera XT kit according to the manufacturer's instructions but excluding the final bead normalization step. Libraries were checked and quantified on an Agilent Bioanalyzer instrument run with an HS DNA kit. Libraries were sequenced on an Illumina MiSeq instrument with 2×250 bp paired-end reads using either V2 or V3 run kits to >25× coverage. Sequence data were uploaded to the European Nucleotide Archive and are available under project accession number PRJEB34817.

### Analysis of sequence data

SNP typing was performed using SAMtools as previously described [23] using the ER Fujisawa strain complete genome sequence [19] as a reference. The predicted effect of each SNP in coding regions was determined using SnpEff 4.3 [24]. Sequence data were assembled using SPAdes 3.13 [25] with the –careful flag active. Draft assemblies were corrected with Pilon 1.21 [26] and annotated using Prokka 1.13.3 with default settings but priority given to protein names in the previously available Fujisawa strain annotation (NCBI Reference Sequence NC_015601.1). Pan-genome analysis was performed using Roary 3.11.2 [27] with a blastp cut-off of 90 %. For testing association between traits and host categories, a subset of 155 diverse isolates were selected based on the SNP data. This was done to reduce bias from possible epidemiological links between isolates from the multiple historical sources contributing isolates to the comparison, e.g. isolates collected during repeated sampling of farms or for outbreak investigation. The reduced set consisted of isolates from pigs (*n*=60), wild boars (*n*=48), wild ungulates (*n*=22), poultry (*n*=13), fish (*n*=4), marine mammals (*n*=4) and other sources (*n*=4). The pan-genome-wide association between each gene in the accessory genome and host of origin for the isolates was tested using Scoary 1.6.16 [28], evaluating pigs and boars together and separately against all other isolate origins. Core genome SNP data were filtered for high-impact changes as defined by SnpEff (loss and gain of stop codons, loss of start codons), summarized to the gene level, converted to a binary matrix (one or more high-impact changes in a given gene or not) and analysed with Scoary in the same way. For both accessory genome and high-impact SNP data, any genes with *P*-value <0.05 after correction for multiple hypothesis testing with Benjamini–Hochberg (BH) method [29] were considered significant. Accessory genome genes were translated with the Sequence Manipulation Suite [30] and protein subcellular localization prediction was performed on the resulting protein sequences using PSORTb 3.0 [31]. Over-/under-representation of localizations between gene categories (genes in the Fujisawa genome, accessory genome genes, significantly host-associated genes) was investigated using Fisher’s exact test with the alternative hypothesis of the true odds ratio not being equal to 1, implemented in R 3.5.0, with any *P*<0.05 considered significant. Antimicrobial resistance genes in pig and boar isolates were detected using ResFinder [32] implemented at the Center for Genomic Epidemiology website (https://cge.cbs.dtu.dk). Surface protective antigen (*spa*) type was determined by comparing six sequences representative of *spaA*, *spaB1*, *spaB2* and *spaC* [33] with all draft assemblies using blast+. The PCR-based typing scheme for serotypes 1a, 1b, 2 and 5 developed by Shiraiwa *et al*. [34] was implemented *in silico* by a blast+ search of all primer sequences with the -task ‘blastn-short’ option active, requiring a full-length hit with 95 % similarity for a positive primer site identification. Isolates that did not match any of the targeted serotypes due to absent or inconsistent results were classified as non-typable (NT). To better visualize the relationship between SNP data, pig/wild boar as host, *in silico* serotype, and the most common antimicrobial resistance genes, a neighbour-joining tree was generated in SplitsTree and used to create a Microreact [35] project containing the pig and wild boar isolates included in the pangenome analysis.

## Results

### SNP clustering and *spa*-typing

**Fig. 1.**
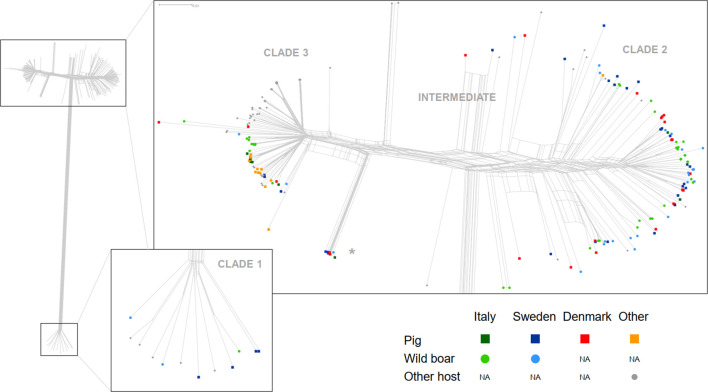
NeighborNet representation of SNP variation among isolates of ER from pigs and boars from Italy, Sweden, Denmark and other countries (*n*=158) and isolates from other hosts including wild and domestic animals (*n*=85). Left side: overview; right side upper panel: detail of clades 2, 3 and intermediate with pig/wild boar isolates highlighted; right side lower panel: detail of clade 1 with pig/wild boar isolates highlighted. A clade consisting of only pig and boar isolates discussed in the main text is marked with an asterisk.

SNP typing revealed pig isolates from Sweden, Italy and Denmark to be phylogenetically diverse in the clade 2 – intermediate-clade 3 spectrum (Fig. 1). The same was true for Swedish and Italian wild boar isolates, which did not cluster separately from pig isolates considering the whole network. There were several examples of smaller clusters of isolates from only pigs, boars, or pigs and boars combined. A distinctly smaller clade of isolates branching off from the main tree near clade 3 consisted of only pig isolates from all three countries and a single boar isolate from Sweden (Fig. 1, asterisk). Clade 1 only included boar isolates from Sweden and Italy together with isolates from healthy Swedish pigs at slaughter, but no pig isolates from animals with known clinical signs of ER infection. All pig and boar isolates in clades 2 and 3 as well as all intermediate isolates carried *spaA*, whereas all clade 1 isolates carried *spaB*.

**Fig. 2.**
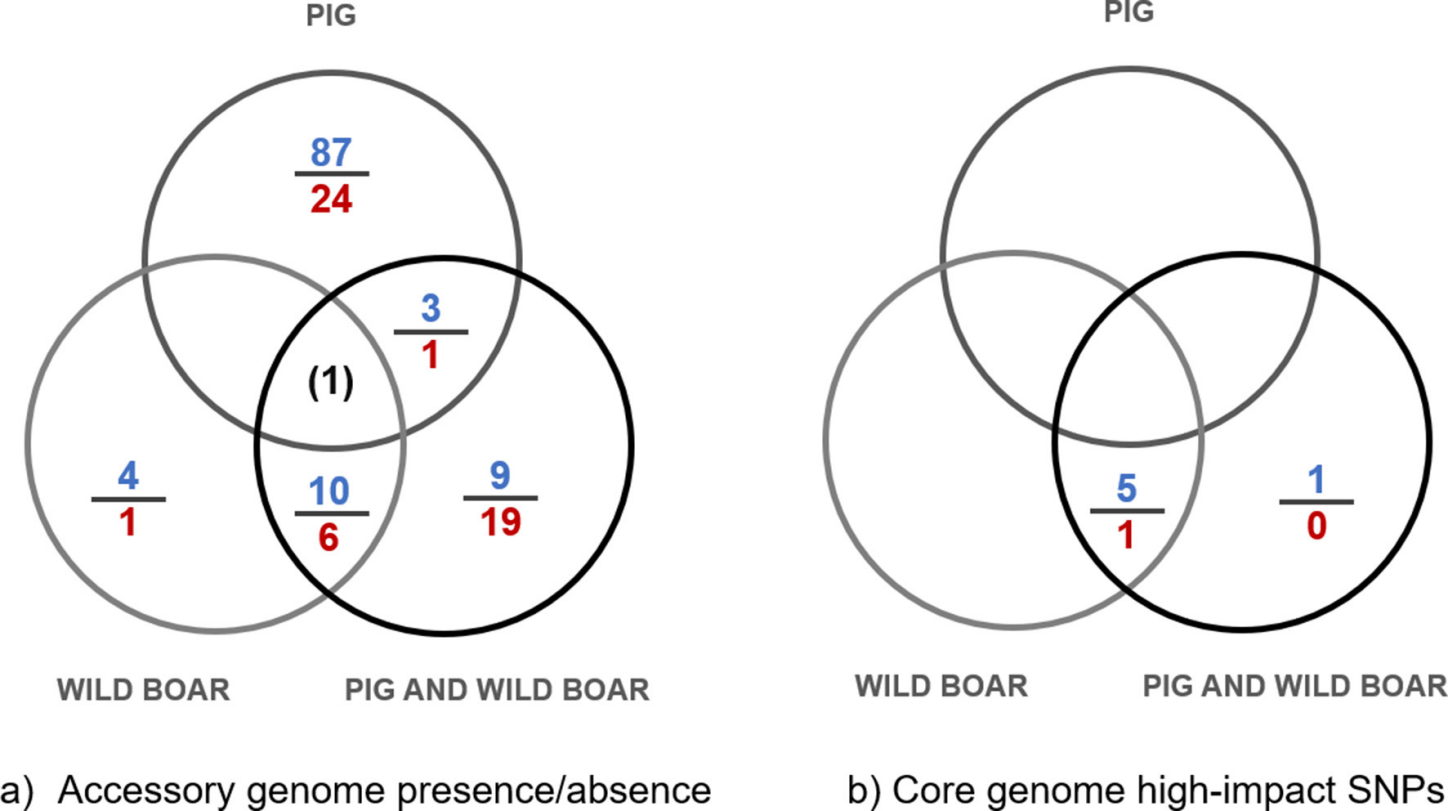
Venn diagram showing the results from Scoary testing of association between host type (pig, wild boar and all other) and (a) the presence or absence of accessory genome components (genes and gene variants), and (b) the presence or absence of high-impact SNPs in core genome genes. For each field, the number of significantly over-represented genes or gene variants (BH-corrected *P*<0.05) is shown in blue over the line and the number of under-represented shown in red under the line. Empty fields indicate no genes were over- or under-represented. A single gene was over-represented in boars but under-represented in two other categories (a, central field).

SnpEff analysis identified 202 high-impact SNPs in 148 different core genome genes; 66 of these genes had high-impact SNPs in more than one isolate. In pigs and wild boars together, loss of function mutations were significantly over-represented, as determined by Scoary, in transferase genes (*nadD*, *patA*), a putative ABC transporter protein (ERH_1125), a sodium-driven multidrug efflux pump (ERH_1256), one of the two types of the 50S L31 ribosomal protein encoded by *rpmE2*, and a short unannotated gene (ERH_0280) (BH-adjusted *P*<0.05). Additionally, a significant correlation was observed with a shorter variant of the gene encoding the alpha subunit of DNA polymerase III (*dnaE*) caused by an alternative/premature stop, which occurred only in a non-clonal set of 15 clade 2 pig and wild boar isolates from Sweden and Italy. Boars analysed alone showed the same set of significant genes as when analysed together with pigs, except for *dnaE*. No high-impact core genome mutations were significantly associated with pigs alone (Fig. 2).

### Pan-genome analysis and association between host type and accessory genome content

**Fig. 3.**
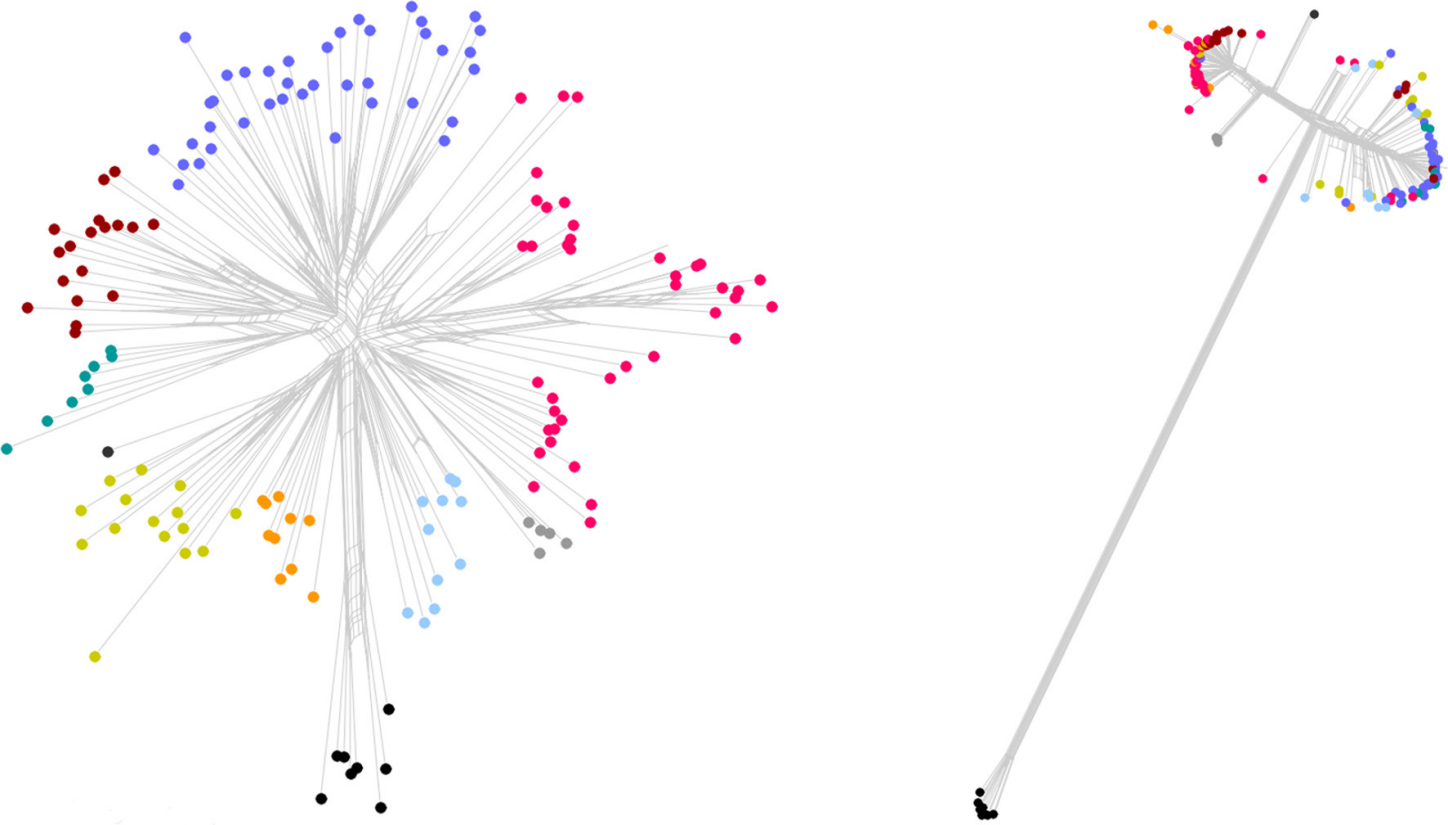
Comparison of NeighborNet representations of the presence or absence of accessory genome components as determined by PanSeq analysis (left) with clusters coloured, and the same clusters projected on the observed SNP variation among the same isolates (right) using the same colour coding.

Pan-genome analysis revealed a set of 1181 core genes or gene variants (present in >99 % of the included genomes) at 90 % sequence similarity, 676 genes present in 15–99 % of isolates and 3057 genes present in <15 %. The pan-genome reference sequences in fasta format are presented in File S1. NeighborNet analysis of presence/absence data for all accessory genes (i.e. non-core) showed strong reticulation and clustering of unrelated isolates as determined by SNP typing, indicating a high degree of horizontal gene transfer in the accessory genome. However, much of the broader clade structure in the SNP data was reflected in the accessory genome content (Fig. 3).

Several genes were found to be significantly associated with host types (Fig. 2, Table S1), of which some were clearly related to mobile genetic elements including transposase and integrase genes. Most genes associated with boars vs. all others were the same as for boars together with pigs; the five genes unique for boars were unannotated or related to mobile elements. In contrast, 111 genes were uniquely correlated to domestic pigs as hosts compared to all others but were not significant when considering boars and pigs together. The majority were either over-represented (*n*=39) or only occurred among pig isolates (*n*=51) (Table S1). Most of these genes lacked annotation, but included multiple LPXTG-motif surface proteins, transcriptional regulators, and genes involved in sugar metabolism and heavy metal tolerance. Notably, as evident in the Scoary analysis most of the genes in the serovar determining region of ER serovars 1 and 2 [36] including *tagD*, CDP-glycerol-poly(glycerophosphate) glycerophosphotransferase, *epsG*, a putative non-coding gene, a gene encoding a protein of unknown function, a sugar transferase, an amino transferase and a polysaccharide biosynthesis protein were significantly associated with the pig host. Of these eight genes, six showed an over-representation among pigs of variants consistent with strains of high virulence for pigs, e.g. gene variants present in Fujisawa (serovar 1) and SE-9 (servovar 2). Two showed under-representation of variants with low sequence similarity to the classical pig pathogenic strains, i.e. variants which were not present in Fujisawa or SE-9, were also rare among pig isolates in general. Consistent with this, a high proportion of pig and wild boar isolates were identified as serotypes 1a, 1b or 2 by *in silico* PCR (Table S2, https://microreact.org/project/wLkvdEnp9iNXMJhGb3bgxZ). Clade 1 isolates and the few isolates from healthy pigs investigated did not belong to serotypes 1 or 2 as determined by *in silico* typing.

Compared to the distribution of predicted subcellular localization for gene products in the Fujisawa reference genome, the accessory genes and gene variants identified by the pan-genome analysis were more likely to be extracellular, to be associated with the cell wall or to have unknown localizations (Table 1). The same trends were observed to an even greater degree for the subset of the accessory genome that had been identified as significantly associated with pigs, boars or both categories of hosts, with particularly high proportions of extracellular and cell wall localization predictions, although these comparisons were based on a much lower number of genes (Table 1). The number of genes with high-impact SNPs was too low for statistical analysis to be meaningful.

**Table 1.** Predicted subcellular localizations of genes in the ER Fujisawa strain reference genome (for comparison), all accessory genes identified in the pangenome analysis, the subset of these genes that were found to be significantly over-/under-represented in isolates from pigs, boars or both, and the subset of core genes with over-/under-representation of high-impact SNPs among isolates from pigs, boars or both Results from Fisher’s exact test comparison with the Fujisawa genome are shown as: ^ns^ *P*>0.05, *P≤0.05, **P≤0.01, and ***P≤0.001; no testing was performed for SNP data due to the low counts.

	Fujisawa genome	Accessory genome	Pig/boar-associated accessory genes	Pig/boar-associated high-impact SNP genes
Total gene count	1697	4933	165	7
**Predicted localization**				
Cell wall	33 (1.9 %)	117 (2.4 %)^ns^	7 (4.3 %)^ns^	0
Cytoplasmic	919 (54 %)	2329 (47 %)***	69 (42 %)**	3 (43 %)
Cytoplasmic membrane	469 (28 %)	1077 (22 %)***	51 (31 %)^ns^	2 (29 %)
Extracellular	10 (0.6 %)	76 (1.5 %)**	4 (2.4 %)*	0
Unknown	266 (15 %)	1334 (27 %)***	34 (21 %)^ns^	2 (29 %)

### Putative genetic determinants of antimicrobial resistance in isolates from pigs and wild boars

One or more of 11 putative genetic resistance determinants were detected in 18/60 (30 %) of investigated pig isolates but only 3/48 (6 %) of wild boar isolates (Table 2). The most common resistance gene was *tet*(M) conferring tetracycline resistance; this was also the only resistance gene to occur in wild boar isolates. The second most common was the combination of *lsa*(E) and *lnu*(B), which co-occurred and in most cases were found on the same contig. Both of these genes confer resistance to lincosamides; *lsa*(E) also confers resistance to pleuromutilin and streptogramin A.

**Table 2.** Presence of genetic antibiotic resistance determinants among pig and wild boar isolates in the present study The first column shows the number of isolates from each host category in each country.

Resistance	Aminoglycosides				Lincosamides, pleuromutilin, streptogramin A	Lincosamides	Macrolides, lincosamides, streptogramin A	Macrolides		Tetracyclines	
Gene	*str*	*aadD*	*ant(9)-la*	*ant(6)-la*	*lsa(E)*	*lnu(B)*	*msr(D)*	*erm(G)*	*erm(T)*	*tet(T)*	*tet(M)*
**Pigs (** ***n*** **=60)**	**2**	**1**	**3**	**3**	**10**	**10**	**1**	**1**	**1**	**1**	**16**
Italy (8)	0	0	0	1	1	1	0	0	0	0	4
Denmark (13)	1	0	1	0	2	2	1	1	0	1	1
Sweden (28)	0	0	0	0	0	0	0	0	0	0	0
Other (11)	1	1	2	2	7	7	0	0	1	0	11
**Wild boars** (48)	**0**	**0**	**0**	**0**	**0**	**0**	**0**	**0**	**0**	**0**	**3**
Italy (25)	0	0	0	0	0	0	0	0	0	0	3
Sweden (23)	0	0	0	0	0	0	0	0	0	0	0

## Discussion

### Genotypes, host bias and virulence in ER

ER can be carried asymptomatically by many animal species and be transmitted via environmental contamination, suggesting that exposure to this species must be relatively common for domestic animals and in most cases does not lead to clinical signs of disease. It is therefore reasonable to assume that the occasional outbreaks are to some extent the result of variable strain-level virulence for a given host combined with the effects of host susceptibility and environmental factors. In the present study we show that certain genetic features – including the presence/absence or substantial sequence variation (i.e. above the pan-genome comparison threshold) in transcriptional regulators, metabolic pathway genes and many currently un-annotated genes – are significantly over-represented in isolates from pigs and wild boars compared to isolates from other sources. Variation among genes producing products destined for the cell wall or for export outside the cell appear to be more likely to be associated with the pig and boar hosts, consistent with direct host–pathogen interaction as a driving force in ER genome evolution. For pigs, certain ER serovars, e.g. 1a, 1b and 2, are widely considered to be more pathogenic whereas other serovars are considered to mostly lack clinical relevance [3]. We show that several genes known to be involved in serovar determination and capsule expression and consistent with serovars associated with severe clinical signs in pigs [36] are indeed more common among pig isolates, and to some extent also among wild boar isolates. As also indicated by *in silico* serotyping, these genetic features appear to be poorly linked to phylogenetic information from SNP typing, occurring in both clades 2 and 3 as well as intermediate isolates. This is consistent with previous studies concluding that ER is a weakly clonal species of generalist phylogenetic lineages [17, 37], and adds further weight to the notion of a significant role for horizontal gene transfer in the evolution of ER. It is thus possible that ER strains form a spectrum of generalist to specialists, with specific host adaptations having evolved independently multiple times in the evolutionary history of the species. A recent study applying whole-genome SNP typing to Japanese isolates from acute cases of erysipelas in pigs showed that the dissemination of multiple clonal lineages of presumably high-virulence ER had resulted in a national increase of serovar 1a outbreaks, indicating that successful specialists can rapidly spread once introduced in a susceptible animal population [20]. In contrast, ER clade 1 has previously been suggested to have a potential link to marine animals [17] and has not been observed in pigs. In the present study we show clade 1 to occur in apparently healthy pigs from Sweden and in apparently healthy wild boars from both Sweden and Italy, but to be absent among isolates from pigs with signs of disease or unknown clinical status. Clade 1 isolates generally carry the *spaB* variant of the surface protective antigen gene of ER, known to be important for host–pathogen interactions, whereas the other clades generally carry *spaA* [17], and as evident in the present study there are substantial differences overall between clade 1 and all other ER in terms of accessory genome content (Fig. 3). None of the clade 1 pig or boar isolates carried any antibiotic resistance genes or genes previously identified as serovar-related [36] found to be over-represented among pigs and wild boars in general. Our findings suggest that carriership of ER clade 1 without clinical signs of disease could be widespread among pigs and perhaps other animals, although the number of clade 1 isolates analysed was limited and further investigation is warranted. This also highlights the more general problem of sampling bias towards strains causing overt disease when performing comparisons to identify phylogenetic and genotypic traits associated with virulence.

### Genotypic differences probably reflect the use of antibiotics and heavy metal supplements in pig farming

Among pig isolates we observed a high prevalence of tetracycline resistance genes, *tet*(M), occuring in the context of Tn916-like conjugative transposons. These mobile genetic elements are known to confer tetracycline resistance in multiple other species of gram-positive bacteria [38] and the *tet*(M) gene itself has been reported to occur in tetracycline-resistant isolates of ER in Japan [39]. We also noted that several pig isolates carried a previously described gene cluster conferring combined resistance to pleuromutilins, lincosamides and streptogramin A (known as the PLS_A_ phenotype) occurring in ER and other bacterial species [40]. However, in contrast to this previous study where the PLS_A_ region and *tet*(M) transposon were located adjacent to each other on the ER chromosome [40] we show that both of these resistance elements occur independently among ER strains. The major indications for antibiotic treatment of pigs in Europe are respiratory tract infections, diarrhoea and *Streptococcus suis* infection, with several classes of antibiotics including macrolides, (fluoro)quinolones and lincosamides used in addition to penicillin and tetracycline [41]. Pleuromutilin is frequently used to treat swine dysentery (i.e. *Brachyspira* infection), with increasing problems with resistance development worldwide [42]. ER is generally sensitive to penicillin antibiotics and this is considered the primary treatment option, but tetracycline added to the water supply is sometimes used to treat larger outbreaks [3]. We can therefore assume that the observed resistance gene distribution among pig ER isolates is the combined result of selective pressure from both treatment of ER and from selection when ER is present as a co-infection to a primary treated pathogen. Conjugative transfer of Tn916 has been shown to be induced by the presence of not only tetracycline but also other antibiotics such as macrolides, lincosamides and streptogramin [43]. Because it is known that a high prevalence of asymptomatic ER infection occurs in pigs [3], ER bacteria can potentially serve as a stable reservoir of resistance genes for other pathogens via horizontal gene transfer. We also note that differing levels of antibiotic treatment of food-producing animals between countries is reflected in differing frequencies of resistance gene occurrence in ER. The use of antibiotics for food-producing animals in Sweden is low [total sales 12 mg/production corrected unit (PCU) in 2016] and largely restricted to penicillins and sulphonamides [44], and no resistance genes were detected among the Swedish pig ER isolates. More antibiotics including tetracyclines and pleuromutilins are used in Denmark and Italy (total sales 40 and 295 mg/PCU in 2016) [44], with correspondingly higher numbers of isolates found with resistance genes (Table 2).

Zinc and copper additives are given to pigs in compound feed to satisfy nutritional requirements, but they also have an antimicrobial effect and are therefore in some cases given in higher doses as an alternative to in-feed antibiotics for growth promotion [45]. This leads to the dissemination of mobile genetic elements conferring heavy metal resistance among the bacterial flora of treated animals [45]. A combination of genes encoding a copper chaperone, a heavy metal translocating P-type ATPase, and a third gene of unknown function occurred only together and was significantly over-represented among pig isolates but not boar isolates in the present study, perhaps representing an example of this process. This gene combination occurred in phylogenetically diverse isolates including all clades and in all three countries. Co-selection for antibiotic resistance and heavy metal resistance has been reported to occur, for example, by co-location of resistance genes on plasmids [45], but there was no evidence of this in the present study as the putative heavy metal resistance genes also occurred in isolates lacking any antibiotic resistance genes.

### Limitations of the present study and future directions

The increasing accessibility of genomic data from large collections of bacterial isolates has created opportunities to better understand the genetic determinants of virulence and host predilection among complex multi-host pathogens such as ER. However, bias in the investigated isolate collections remains a major limitation of such studies, as systematic collection is costly and can rarely be standardized and coordinated across multiple countries, isolation sources and for long time periods, as would be ideal. The present study included a substantial panel of ER isolates from pigs and wild boars collected in multiple countries, but no metadata in terms of, for example, disease severity were available for most isolates. Although we note marked differences between isolates from healthy and diseased pigs, this is based on a limited number of isolates from only Swedish healthy pigs, as such sampling is rarely performed. A similar limitation is the absence of wild boar isolates from animals with clinical signs of infection, which are difficult to collect. We must therefore expect a degree of conflation between the effects of host bias and relative virulence in our comparisons, perhaps reducing the power to identify traits related to both. Despite these limitations, the presented analysis is the first systematic attempt to compare pig and wild boar isolates to each other as well as to those from other sources. We note that without any a priori assumptions our analysis indicates serovar-associated genes as linked to the pig host, which is in concordance with previous observations [36]. The differences in, for example, the frequency of antibiotic resistance genes between domestic pig and wild boar isolates indicates that transmission of adapted strains may occur to a limited degree between the two hosts; targeted local sampling of wild boars living in the vicinity of specific pig farms would perhaps give a more detailed picture in this regard as our comparison is on the national and regional levels. The number of isolates from other relevant host species such as poultry was also low and unbalanced in the present study. Future comparisons including more poultry isolates will be necessary to better understand the links between bacterial genomics and host factors in the development of clinical erysipelas.

## Supplementary Data

Supplementary material 1
